# The N-terminal portion of autoinhibitory element modulates human endothelial nitric-oxide synthase activity through coordinated controls of phosphorylation at Thr^495^ and Ser^1177^

**DOI:** 10.1042/BSR20140079

**Published:** 2014-08-06

**Authors:** Pei-Rung Wu, Bo-Rui Chen, Chi-Chun Hsieh, Wei-Chung Lin, Kenneth K. Wu, Yeukuang Hwu, Pei-Feng Chen

**Affiliations:** *Institute of Cellular and System Medicine, National Health Research Institutes, Zhunan, Miaoli County 35053, Taiwan; †Institute of Physics, Academia Sinica, Nankang, Taipei 11529, Taiwan; ‡The Genomics Research Center, Academia Sinica, Taipei 11529, Taiwan; §Quality control Department, Adimmune Corporation, Taichung 42743, Taiwan; ∥New Product Integration, AU Optronics Corporation, Taichung 40763, Taiwan; ∥Metabolomic Medicine Research Center, China Medical University, Taichung 40402, Taiwan

**Keywords:** autoinhibitory element, calcium/calmodulin, CaM-binding domain, endothelial nitric-oxide synthase, mobility shift gel, phosphorylation/dephosphorylation, AIE, autoinhibitory element, CaM, calmodulin, CBD, CaM-binding domain, DMEM, Dulbecco’s modified Eagle’s medium, EGFP, enhanced green fluorescent protein, eNOS, endothelial nitric-oxide synthase, H_4_B, (6R)-5,6,7,8-tetrahydro-L-biopterin, GS, goat serum, HEK-293, human embryonic kidney 293 cell, heNOS, human eNOS, iNOS, inducible NOS, Myr^−^eNOS, myristylation-deficient eNOS, nNOS, neuronal NOS, WTeNOS, wild-type eNOS

## Abstract

NO production catalysed by eNOS (endothelial nitric-oxide synthase) plays an important role in the cardiovascular system. A variety of agonists activate eNOS through the Ser^1177^ phosphorylation concomitant with Thr^495^ dephosphorylation, resulting in increased ·NO production with a basal level of calcium. To date, the underlying mechanism remains unclear. We have previously demonstrated that perturbation of the AIE (autoinhibitory element) in the FMN-binding subdomain can also lead to eNOS activation with a basal level of calcium, implying that the AIE might regulate eNOS activation through modulating phosphorylation at Thr^495^ and Ser^1177^. Here we generated stable clones in HEK-293 (human embryonic kidney 293) cells with a series of deletion mutants in both the AIE (Δ594–604, Δ605–612 and Δ626–634) and the C-terminal tail (Δ14; deletion of 1164–1177). The expression of Δ594–604 and Δ605–612 mutants in non-stimulated HEK-293 cells substantially increased nitrate/nitrite release into the culture medium; the other two mutants, Δ626–634 and Δ1164–1177, displayed no significant difference when compared with WTeNOS (wild-type eNOS). Intriguingly, mutant Δ594–604 showed close correlation between Ser^1177^ phosphorylation and Thr^495^ dephosphorylation, and NO production. Our results have indicated that N-terminal portion of AIE (residues 594–604) regulates eNOS activity through coordinated phosphorylation on Ser^1177^ and Thr^495^.

## Abstract 2

Our findings demonstrate for the first time that AIE insert exerts its regulatory function by coordinate phosphorylation on eNOS Ser^1177^ and Thr^495^, highlighting the importance of AIE in regulating agonist-induced eNOS activation.

## INTRODUCTION

NO generated by eNOS (endothelial nitric-oxide synthase) plays critical roles in cardiovascular biology [1]. eNOS [2] has two other structurally related NOS isoforms-nNOS (neuronal NOS) [3] and macrophage iNOS (inducible NOS) [4]. All three isoforms function as homodimers, with each monomer containing a C-terminal reductase domain and an N-terminal oxygenase domain [5,6]. The reductase domain contains the FMN and FAD and the NADPH-binding site, whereas the oxygenase domain contains protoporphrin IX haeme, H_4_B [(6R)-5,6,7,8-tetrahydro-L-biopterin], zinc, as well as the L-arginine-binding site [7–10]. These two domains are connected by a CaM (calmodulin)-binding site, which is constantly bound by CaM in iNOS in the resting state, whereas CaM binding to nNOS and eNOS requires an elevation of intracellular calcium [11,12].

CaM regulates all three NOS isoforms. The difference in calcium/CaM sensitivity between eNOS (and nNOS) and iNOS has been attributed to two inserts present in the eNOS reductase domain, but absent in the iNOS counterpart. These two inserts, the AIE [autoinhibitory element; residues 594–645 in the FMN-binding domain of heNOS (human eNOS)] and the C-terminal tail, are believed to keep eNOS inactive in resting cells [13,14]. By binding to the CBD (CaM-binding domain) of eNOS, calcium/CaM is postulated to displace these inserts on eNOS, thus facilitating the NADPH-dependent electron flux from the flavins of one subunit to the haeme of the adjacent subunit [15,16]. Deletion of either the AIE or the C-terminal tail has been consistently associated with reduced calcium/CaM dependence of NO production, underscoring the importance of these two inserts in mediating eNOS activation by calcium/CaM [17–19].

The regulation of eNOS catalysis was originally thought to be a simple calcium/CaM-dependent process. However, there is increasing evidence that eNOS activity is tightly controlled by a number of regulatory mechanisms, including multi-site phosphorylation, the autoregulatory property of the eNOS itself, protein–protein interactions and subcellular localization [20]. In endothelial cells, heNOS can be phosphorylated at Ser^114^, Thr^495^, Ser^615^, Ser^633^ and Ser^1177^. Phosphorylation of eNOS at Ser^615^, Ser^633^ and Ser^1177^ enhances the interaction between eNOS and calcium/CaM, resulting in eNOS activation at lower calcium concentration, whereas phosphorylation of Thr^495^ (within the CBD) impedes CaM-binding and enzyme activation [21–24]. Diverse stimuli, including mechanical forces, humoral factors and pharmacological agonists, have been reported to activate eNOS through Ser^1177^ phosphorylation concurrent with the dephosphorylation of Thr^495^. The coupling change of the phosphorylation state at these two residues has been indicated to be central in the mechanism of calcium/CaM-dependent eNOS regulation [25,26]. However, the mechanism by which the eNOS activation is associated with the increased Thr^495^ dephosphorylation and Ser^1177^ phosphorylation remains poorly elucidated.

The eNOS is regulated by a variety of post-translational events and emerging evidences imply that each regulatory mechanism is closely interconnected [27]. The crystal structure of the nNOS reductase domain, along with perturbation studies of either the AIE or the C-terminal tail, suggests the possibility of direct interactions between the AIE and the C-terminal tail, and between the AIE and CaM [10,12,28]. As Thr^495^ is located within the CaM-binding region and Ser^1177^ is positioned in the C-terminal tail, we propose that the AIE enables eNOS regulation through the modulation of eNOS phosphorylation at Thr^495^ and Ser^1177^. To test this hypothesis, we generated deletion mutants in both the AIE (Δ594–604, Δ605–612 and Δ626–634) and the C-terminal tail (Δ14). We then established stable clones in HEK-293 cells with these eNOS constructs and studied how the deletions affected NO production and the phosphorylation state in eNOS. Our findings demonstrate that the N-terminal portion of the AIE is crucial for the control of eNOS phosphorylation at Thr^495^ and Ser^1177^.

## EXPERIMENTAL

### Materials

L-[2,3,4,5-^3^H]arginine (NET1123250UC) was obtained from PerkinElmer Life Sciences. H_4_B was purchased from Research Biochemical International. Bradford protein dye reagent and electrophoretic chemicals were acquired from Bio-Rad. Restriction enzymes were purchased from New England Biolabs. pCDNA3.1(+) was obtained from Life Technologies. pEGFP (enhanced green fluorescent protein)-N1 was purchased from Clontech/Takara. HEK-293 cells (CRL-1573) were obtained from A.T.C.C. Antibodies specific to the eNOS phosphorylation sites at Ser^1177^ and Thr^495^ were purchased from EMD Millipore Co. Antibodies specific to the eNOS phosphorylation sites at Ser^114^, Ser^615^ and Ser^633^ were obtained from Upstate Biotechnology. Anti-eNOS monoclonal antibody was purchased from BD Transduction Laboratories. Anti-actin was obtained from Millipore. The fluorescein isothiocyanate-labelled anti-mouse secondary antibody was purchased from Jackson ImmunoResearch Laboratories. The Dowex AG 50W-X8 (cation-exchange resin), NADPH and all other reagents were acquired from Sigma-Aldrich Co.

### Generation of eNOS constructs

**Figure 1:**
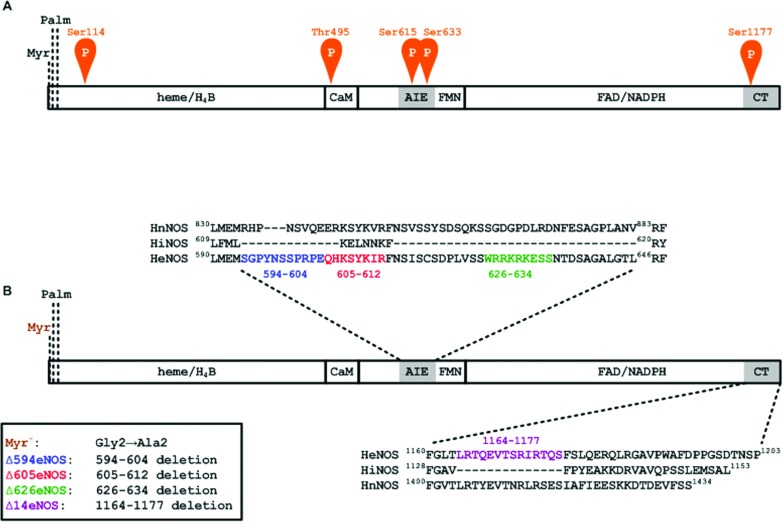
heNOS and the deletion constructs (**A**) The relative positions of phosphorylation sites in heNOS. Blocks indicate the binding sites for haeme, H_4_B, CaM, FMN, FAD and NADPH in eNOS. AIE and CT refer to the AIE and the C-terminal tail, respectively. The myristoylation site (Myr.) and palmitoylation sites (Palm.) are noted. Potential phosphorylation sites Ser^114^, Thr^495^, Ser^615^, Ser^633^ and Ser^1177^ are indicated. (**B**) Constructs for the eNOS deletion mutants. The sequences of the AIE and the C-terminal tail are shown above and below, respectively. The Δ594 is a deletion of the AIE region encompassing residues Ser^594^–Glu^604^ (shown in blue); Δ605 is a deletion of the AIE region encompassing residues Gln^605^–Arg^612^ (shown in red); Δ626 is a deletion of the AIE region encompassing residues Trp^626^–Ser^634^ (shown in green); Δ14 is a deletion of 14 amino acid residues at the C-terminal tail encompassing residues Leu^1164^–Ser^1177^ (shown in violet).

A cytosolic eNOS was generated by altering the glycine at position 2 of WTeNOS (wild-type eNOS) to alanine (Myr^−^eNOS) as previously described [29,30]. The Myr^−^eNOS and WTeNOS cDNAs were subcloned into the mammalian expression vector pCDNA3.1(+) (Accession K03104) through EcoRI restriction enzyme digest. We previously constructed four cytosolic forms of eNOS mutants with deletions from the AIE (∆594–604, ∆605–612 and ∆626–634) and the C-terminal tail (∆14, residues 1164–1177) in pVL1392 baculoviral expression vector [19,31]; the deleted amino acid sequences for each mutant are shown in Figure 1. To create the membrane-bound mutants, the mutated regions were generated by PCR using the cytosolic form of the respective mutants in pVL1392 expression vector as a template. The primers 5′-CTCGAACACGAGACGCTGGTGCT-3′ and 5′-CTGCTCTACTGCCACGGGCTC were used for amplification of ∆594–604, ∆605–612 and ∆626–634. The amplified fragments were subcloned to replace the corresponding fragment in WTeNOS–pCDNA3.1(+) through BstEII and SgrAI restriction enzyme digest sites. The primers 5′-CTGCGGAGGTGCACCGCGTGC-3′ and 5′-CACAGTCGAGGCTGATCAGC were used for the amplification of ∆14, and the amplified fragment was used to replace the corresponding fragment in WTeNOS-pCDNA3.1(+) through the XhoI restriction enzyme digest site.

The various eNOS–EGFP pcDNA3 plasmids were generated by fusing pEGFP-N1 (Accession #U55762) directly after the final amino acid of the C-terminus of eNOS by the standard two-stage PCR cloning methods. The pEGFP-N1 and eNOS (WTeNOS, Myr^−^eNOS, ∆594, ∆605, ∆626 and ∆14 in pVL1392) [19,31] were used as templates for PCR. The first set of primers 5′-CGAGACGCTGGTGCTGGTGGTAAC-3′ (sense) and 5′-GGGGCTGTTGGTGTCTGAGCCG-3′ (antisense) was used to amplify eNOS. The second set of primers 5′-**p**GTGAGCAAGGGCGAGGAGCTGTTC-3′ (sense) and 5′-CTGATTATCATCTAGAGTCGCGGCCGCTTTAC-3′ (antisense) was used to amplify the EGFP fragment. We phosphorylated the EGFP sense primer (p in boldface) and fused these two fragments by the blunt-end ligation. The eNOS-EGFP fragments were subcloned to replace the corresponding fragment in WTeNOS–pCDNA3.1(+) or Myr^−^eNOS–pCDNA3.1(+) through BstEII and XbaI restriction enzyme digest sites. The sequences of each construct were confirmed by the Sequencing Service in the core facility of NHRI at Miaoli, Taiwan. The fusion proteins with the expected molecular masses were verified by Western blotting using an anti-eNOS antibody.

### Generation of stable cell lines and fluorescence microscopy

HEK-293 cells were grown in DMEM (Dulbecco's modified Eagle's medium) with 10% (v/v) FBS (Thermo Scientific Hyclone Co.). All eNOS constructs in the pCDNA3.1(+) vector were linearized with PvuI and transfected to HEK-293 cells using *Trans*IT®-2020 (Mirus) according to the manufacturer's instructions. Transfected cells were cultured in the selection medium (DMEM with 10% FBS plus 1000 μg/ml G418). G418-resistant colonies were isolated via limiting dilution and expanded in the selection medium. Stable clones in HEK-293 cells with each eNOS construct were grown in phenol red-free DMEM with 10% FBS plus 500 μg/ml G418 at cell density (3.5×10^4^/cm^2^). The localization of eNOS and eNOS–EGFP fusions in living and fixed cells was examined by fluorescence microscopy 48 h after plating.

To confirm the subcellular location of untagged eNOS constructs, the protein expression of HEK-293 stable clones with eNOS variants was detected with an anti-eNOS antibody in fixed cells. The HEK-293 cells were fixed by 4% (v/v) paraformaldehyde in ice-cold PBS for 15 min supplemented with 5% (v/v) GS (goat serum), subsequently permeabilized with 0.1% (v/v) Triton X-100 in PBS/5% GS for 10 min and blocked with 5% GS in PBS for 30 min. Cells were incubated with anti-eNOS antibody in PBS supplemented with 5% GS at room temperature (25°C) for 1 h, followed by washing and incubation for 30 min with fluorescein isothiocyanate-labeled anti-mouse (diluted 1:100) secondary antibody. After washing, slides were mounted with Slowfade (Life Technologies) and the cells were observed with a fluorescence microscope.

### Determination of nitrate/nitrite in culture medium

Stable clones of eNOS constructs in HEK-293 cells were grown in the phenol red-free selection medium at a cell density of 3.5×10^4^/cm^2^. The medium was collected 48 h after plating, and the nitrate/nitrite accumulation in the culture medium was determined using a colorimetric assay kit according to the manufacturer's instructions (Cayman Chemical Co.). The cells were scraped into a buffer containing 25 mM Tris/HCl, pH 7.5, 0.1 mM DTT (dithiothreitol), 0.1 mM EDTA, 0.1 mM EGTA, 1 μM pepstatin A, 1 μM leupeptin, 1 μM antipain and 10% (v/v) glycerol (Buffer A) at a density of ~1×10^7^ cells/ml for a citrulline formation assay.

### Citrulline formation in disrupted cell preparations

The harvested eNOS–HEK-293 cells were subjected to sonication with 3×1 s bursts using a Tomy UD-200 ultrasonic disruptor (setting 3). The cell lysates were subsequently incubated in Buffer A containing 300 μM CaCl_2_, 100 μM β-NADPH, 10 μM BH_4_, 5 μM L-arginine, and 1 μCi of L-[^3^H]arginine at 37°C for 4 min. The reactions were terminated by ice-cold stop buffer (20 mM HEPES, 2 mM EGTA and 2 mM EDTA, pH 5.5) and then the mixtures were passed through a Dowex AG 50W-X8 (Na^+^ form) column. L-[^3^H]citrulline was quantitated by liquid scintillation counting. An assay in the absence of CaM was carried out to serve as baseline reference.

### Phosphosite-specific antibody analyses

Stable clones of eNOS constructs in HEK-293 cells were grown in the selection medium at a cell density of 3.5×10^4^/cm^2^. After 48 h, the cells were washed twice in ice-cold PBS, pelleted and solubilized in ice-cold lysis buffer containing 50 mM Tris–HCl, pH 7.4, 150 mM NaCl, Halt Protease and Phosphatase Inhibitor Cocktail, (Thermo Scientific Co.), 1% (v/v) Triton X-100 and 10% glycerol for 15 min. The lysates were then clarified by centrifugation. After determining the protein concentrations with a Bradford assay [32], equal amounts of protein were separated on a SDS–PAGE (7.5% gel) [33] (30 μg per lane) and then transferred to a PVDF membrane (Millipore Co.) overnight at 4°C and 35 V. eNOS phosphorylation at Ser^114^, Thr^495^, Ser^633^, Ser^615^ and Ser^1177^ was detected using commercially available phosphosite-specific antibodies. The membrane was exposed to the primary antibody overnight at 4°C, washed, incubated with a secondary antibody conjugated to horseradish peroxidase, washed again, and then developed using a chemiluminescent reagent (Pierce). To ensure the consistent protein loading and the equivalent expression of the eNOS constructs in HEK-293 cells, the same membrane was stripped and probed against an eNOS monoclonal antibody. Densitometric analyses on the Western blots were performed using a Gel-Pro Analyzer system.

### CaM mobility shift gels

Several peptides representing the heNOS CBD with point mutations at putative phosphorylation site, Thr^495^ were synthesized by Peptide 2, Inc. The peptide sequences based on heNOS numbering 491–510 are: (i) Unmodified CBD, T495: TRKK**T^495^**FKEVANAVKISASLM; (ii) phospho-null CBD, A495: TRKK**A^495^**FKEVANAVKISASLM; (iii) phosphomimetic CBD, D495:TRKK**D^495^**FKEVANAVKISASLM and (iv) phosphorylated CBD, pT495: TRKK**pT^495^**FKEVANAVKISASLM

Human CaM was purified essentially as described previously [34]. CaM (200 pmol) was incubated with each peptide at several molar ratios for 1 h in 10 μl of buffer containing 20 mM Tris, pH 7.5 with 100 μM CaCl_2_. The sample was subjected to non-denaturing, non-reducing PAGE (18% gel) [33] at 30 mA under high Ca^2+^ conditions (100 μM free Ca^2+^ in all gel buffers). Binding of peptides to CaM was visualized by Coomassie Brilliant Blue R-250 staining. The relative amount of CaM on the gel at each peptide concentration (I) was determined by densitometry and normalized to CaM in the absence of peptide (I_0_). The data are plotted as the mean I/I_0_±S.D. (n=4) versus peptide:CaM ratio.

### Analysis of NOS activity

The expression and purification of eNOS from Sf21 cells was carried out essentially same as previously reported [34]. The inhibition of eNOS oxygenase activity by unmodified or modified CBDs was tested at 37°C for 4 min by measuring L-[^3^H]citrulline formation in a mixture containing 25 mM Tris, pH 7.5, 100 mM NaCl, 0.5 μM CaM, 0.2 mM EDTA, 0.3 mM CaCl_2_, 100 μM β-NADPH, 10 μM H_4_B, 20 μM L-arginine, 1 μCi of L-[^3^H]arginine, 100 nM eNOS, and in the presence or absence of 10 μM of each peptide. Cytochrome *c* reduction was determined at room temperature (25°C) in a reaction mixture containing 25 mM Tris–HCl, pH 7.5, 100 mM NaCl, 10% glycerol, 50 μM cytochrome *c*, 0.5 μM CaM, 100 μM CaCl_2_ and 100 μM β-NADPH in the presence or absence of 10 μM of each peptide. The reaction was initiated by addition of 50 nM eNOS and monitored at 550 nm in a GE geneQuant100 spectrophotometer. Activity was determined using a ΔE_550_=21 mM^−1^cm^−1^. An assay in the absence of CaM was carried out to serve as baseline reference.

### Statistical analysis

The data are expressed as the mean±S.D. of at least three different experiments. Differences were assessed using two types of Student's independent or paired *t* tests. Values of *P*<0.05 were considered statistically significant. All analyses were performed using the SlideWrite Plus software program Version 6.0 for Windows (Advanced Graphics Software, Inc.).

## RESULTS

### Phosphorylation state of eNOS is affected by subcellular localization in HEK-293 cell lines

**Figure 2:**
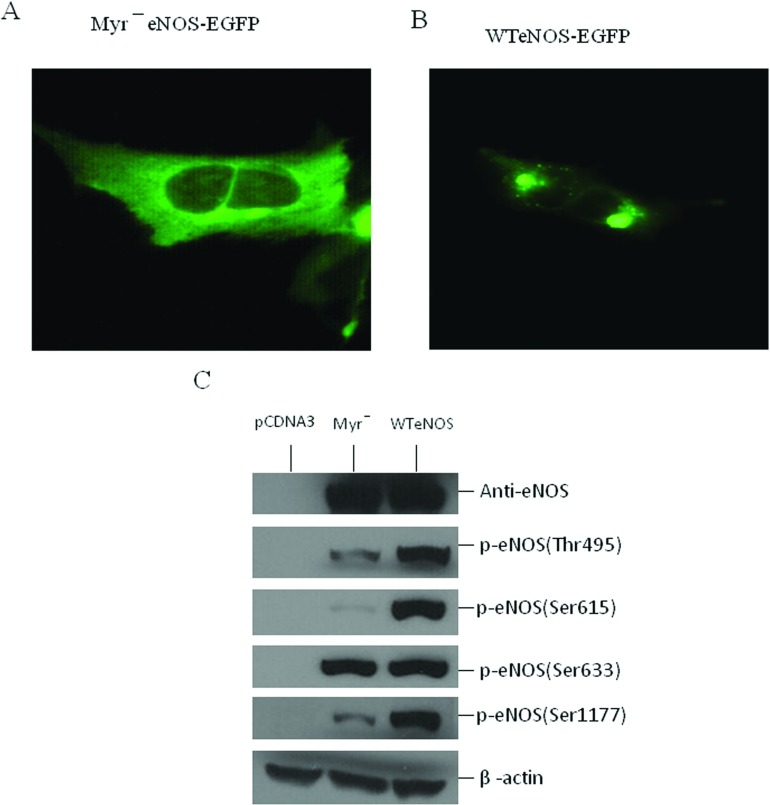
Relationship between the subcellular localization and phosphorylation state of eNOS Stable HEK-293 cell lines with (**A**) myr^−^eNOS-EGFP or (**B**) WTeNOS–EGFP were visualized in live cells by fluorescence microscopy. (**C**) cell lysates from HEK-293 cells transfected with empty vector (pCDNA3), Myr^−^eNOS or WTeNOS were immunoblotted for changes in phosphorylation state of Thr^495^, Ser^615^, Ser^633^ and Ser^1177^. The expression of eNOS and β-actin was used as internal loading control. Western blots shown are the representatives of at least three independent studies.

The WTeNOS is a membrane-associated enzyme via N-terminal myristoylation of the glycine at position 2 [35], which is required for subsequent palmitoylation of the cysteines at positions 15 and 26 (Figure 1) [36,37]. Mutation of Gly-2 to Ala creates a Myr^−^eNOS (myristylation-deficient eNOS), which converts the membrane-associated eNOS to a cytosolic form [29]. The degree and pattern of eNOS phosphorylation have been noted to vary with subcellular location in COS-7 and bovine aortic endothelial cells [38,39]. To investigate whether HEK-293 cell behaves like other cell types with respect to eNOS localization and phosphorylation, stable clones of HEK-293 cells with untagged or tagged constructs of WTeNOS and Myr^−^eNOS were generated. EGFP can be expressed as fusion proteins in a variety of living cells for tracking individual proteins [40,41]. As such, we attached EGFP to the C-terminus of WTeNOS and Myr^−^eNOS (WTeNOS–EGFP or Myr^−^eNOS–EGFP). The subcellular localization of stable clones in HEK-293 cells with WTeNOS–EGFP and Myr^−^eNOS–EGFP constructs was examined by fluorescence microscopy. The Myr^−^eNOS–EGFP evenly distributed fluorescence throughout the cell (Figure 2A), indicating a cytosolic expression. In contrast, WTeNOS–EGFP was primarily localized to the perinuclear region and a small portion was visualized in discrete regions of the plasma membrane [42] (Figure 2B). To confirm the identical localization of the eNOS–EGFP to that of untagged eNOS, HEK-293 stable clones with untagged eNOS constructs were probed with anti-eNOS antibody in fixed cells. The eNOS–EGFP was similar to the untagged eNOS in fixed HEK-293 cells, indicating that EGFP tag did not alter its localization (results not shown). Therefore Gly-2 as previously reported was critical for eNOS membrane association [29,35].

The subcellular localization determined for the WTeNOS and Myr^−^eNOS in HEK-293 cells is comparable with the previous reports in different cell types [39,42]. We thus evaluated the phosphorylation state of these two constructs stably expressed in HEK-293 cells. Several phosphorylation sites have been reported in eNOS, including Ser^1177^, Thr^495^, Ser^114^, Ser^615^ and Ser^633^ (using the human numbering, Figure 1). When the cell lysates of non-stimulated HEK-293 cells stably expressing WTeNOS or Myr^−^eNOS constructs were probed with phosphosite-specific antibodies, we saw a striking decrease in Thr^495^ and Ser^1177^ phosphorylation, nearly no Ser^615^ phosphorylation, and a small decrease in Ser^633^ phosphorylation on the Myr^−^eNOS compared with the WTeNOS (Figure 2C), reflecting that subcellular localization influenced the eNOS phosphorylation state in HEK-293 cells. The pattern and degree of phosphrylation vary among these residues, indicating that each phosphorylation site of eNOS may have discrete roles in response to the agonist-elicited eNOS activation process. The commercially available antibody for eNOS phosphosite-Ser^114^ gave a strong non-specific band in the Western blot analysis, so that this antibody was not used for additional experiments (results not shown). To verify the proper expression and equivalent loading of these two constructs, the cell lysates were analysed using eNOS and β-actin antibodies as internal controls. The result shows that the pattern and degree of eNOS phosphorylation in HEK-293 cells with WTeNOS or Myr^−^eNOS construct are consistent with the earlier reports in COS-7 cell and bovine aortic endothelial cell [38,39]. From this analysis, HEK-293 cells that do not express the detectable amount of eNOS can be used as a cellular model for further study in defining the relationship between the AIE and the phosphorylation state at Thr^495^ and Ser^1177^.

### HEK-293 cell lines stably expressing WTeNOS and mutant eNOSs

Myr^−^eNOS stays cytosolic and releases less NO in response to cellular agonists but *in vitro* activity assay under the maximal substrate and cofactors is indistinguishable from WTeNOS [38,39]. Therefore the generation of a soluble Myr^−^eNOS will allow protein purification without detergent solubilization from cells [29,30]. To facilitate eNOS purification, our previous constructs with deletions of the AIE (Δ594–604, Δ605–612, Δ626–634) and the C-terminal tail (Δ14) were created in myristoylation-deficient form [19,31]. As subcellular localization had an effect on the phosphorylation state in HEK-293 cells, we generated in membrane-bound forms rather than soluble forms as in our previous study [19,31]. We subsequently used these membrane-bound eNOS mutant constructs to generate the stable clones in HEK-293 cells. Eight stable clones for each construct were examined using Western blot analyses to ensure the stable and equivalent eNOS expression. Three stable lines of each construct were used in this study. Stable clones transfected with pcDNA3.1(+) empty vector were used as a control in each experiment. The subcellular localization of eNOS mutant proteins in various eNOS–HEK-293 stable clones was confirmed by the immunofluorescence staining. The results showed that these deletion mutants, similar to WTeNOS were mainly located at the perinuclear region (results not shown) as previously reported [42].

### NO_2_^−^/NO_3_^−^ accumulation in eNOS–HEK293 cell culture medium

**Figure 3:**
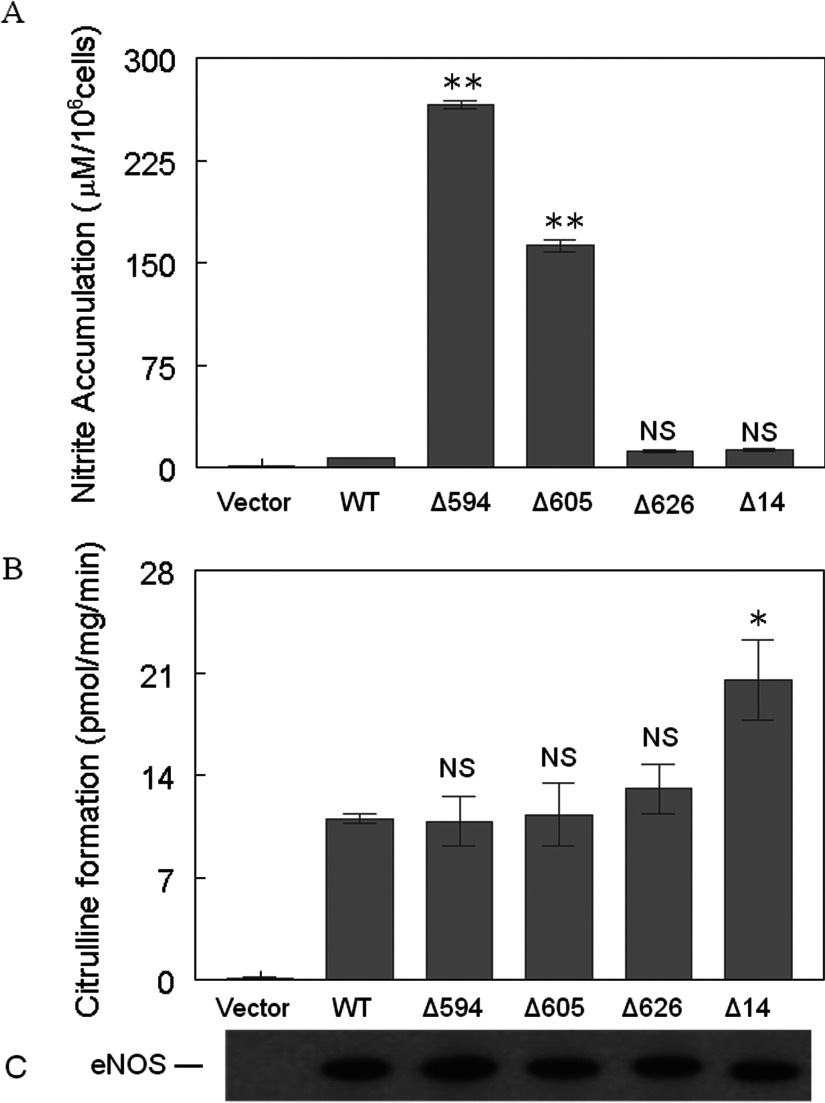
Enzymatic activities of various eNOS—HEK-293 stable clones (**A**) Nitrate/nitrite accumulation in HEK-293 cell culture medium 48 h after plating. The nitrate/nitrite accumulation in culture medium was determined using a colorimetric assay kit obtained from Cayman Chemical Co. (**B**) L-citrulline formation activity was determined in the cell lysates of various eNOS–HEK-293 stable clones. Reactions were carried out as described in the ‘Materials and Methods’ section. (**C**) The eNOS–HEK-293 cell lysates were immunoblotted with an antibody specific to total eNOS to confirm the equal expression levels of each eNOS construct. Data shown are the mean±S.D. (*P<0.05; **P<0.01; NS, no significant difference *versus* WT eNOS). Each experiment was performed in triplicate and repeated three times.

To determine whether these eNOS mutant constructs could produce NO under basal conditions, we collected cell culture medium and measured NO_2_^−^/NO_3_^−^ accumulation in non-stimulated eNOS-HEK-293 cells 48 h after plating. As expected, minimal amounts of NO_2_^−^/NO_3_^−^ were detected in the culture medium from vector or WTeNOS–HEK-293 cells. Interestingly, the deletion of residues 594–604 or 605–612 resulted in a substantial increase in NO_2_^−^/NO_3_^−^ accumulation, to ~160–260 μM per 10^6^ cells, in the culture medium, whereas the deletion of residues 626–634 or 1164–1177 only produced a small increase in NO_2_^−^/NO_3_^−^ accumulation (Figure 3A). These results suggest that Δ594–604 and Δ605–612 are constitutively active and can produce NO under basal conditions. Next, we wanted to determine whether these eNOS mutants were able to produce NO when the disrupted cell lysates were provided with optimal cofactors and substrate, such as Ca^2+^/CaM. L-citrulline formation assays using various eNOS–HEK-293 cell lysates revealed that all these mutants are at least as active as WTeNOS (Figure 3B). Western blot analyses showed that all eNOS–HEK-293 cells had comparable eNOS expression levels (Figure 3C). These results indicate that the differences in NO_2_^−^/NO_3_^−^ accumulation were not because of differences in protein expressions or global conformational changes in various eNOS constructs.

### Phosphorylation state of stably expressed eNOS variants in HEK-293 cells

**Figure 4:**
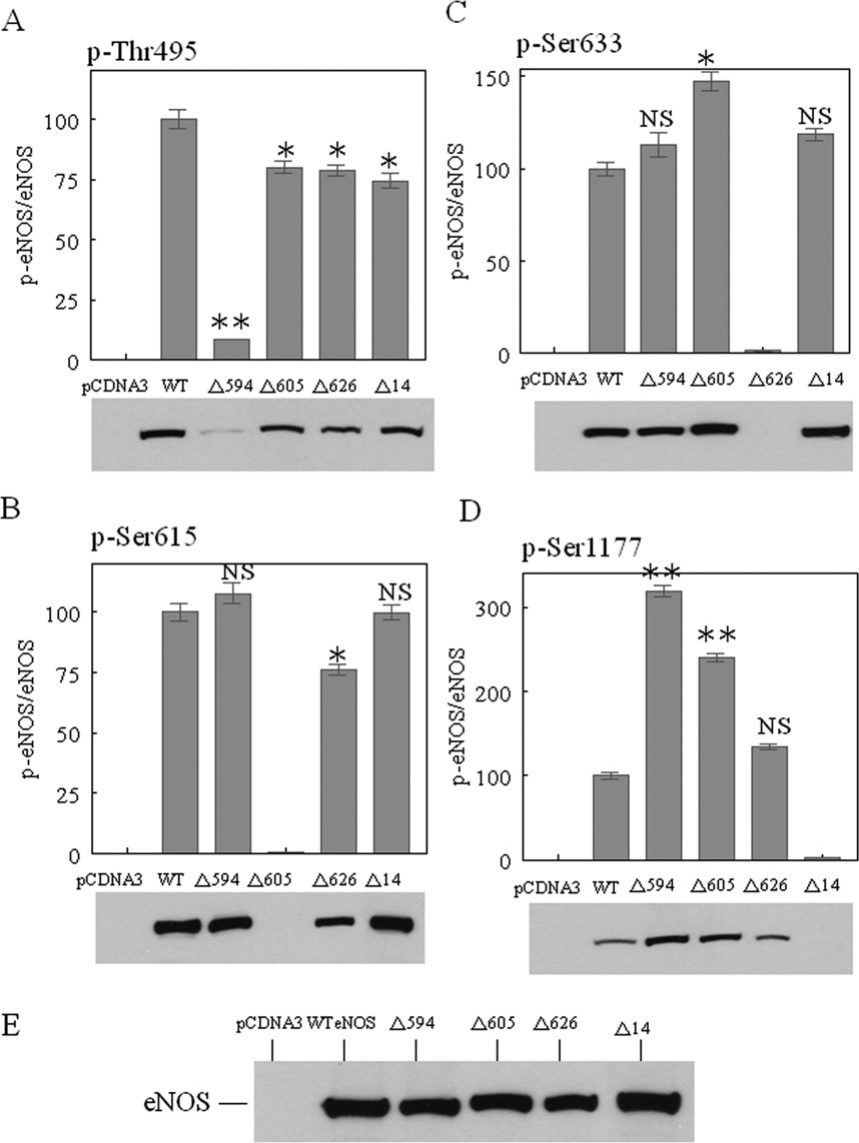
The effects of deletion of the AIE and the C-terminal tail on eNOS phosphorylation The cell lysates from various eNOS–HEK-293 stable clones, including empty pCDNA3.1(+), WT eNOS (WT), Δ594–604 (Δ594), Δ605–612 (Δ605) and Δ1164–1177 (Δ14) were immunoblotted with antibodies specific to phosphosites at (**A**) p-Thr^495^, (**B**) p-Ser^615^, (**C**) p-Ser^633^ and (**D**) p-Ser^1177^. (**E**) An antibody specific to total eNOS was used to confirm equal expression levels for the various eNOS constructs. The blots shown are representative of at least three experiments. Densitometry was used to quantify the phosphorylated eNOS relative to total eNOS of respective constructs. Data are presented as the percentage of WTeNOS and represent means±S.D. (*P<0.05; **P<0.01; NS, no significant difference *versus* WT eNOS).

Since the deletion of residues 594–604 and 605–612 increased NO production in the absence of extracellular calcium (Figure 3A), we hypothesized that mutant eNOS phosphorylation might also be altered. To test our hypothesis, we examined eNOS phosphorylation in various eNOS–HEK-293 stable clones under basal conditions. Immunoblotting with phosphosite-specific eNOS antibodies revealed no Ser^615^ phosphorylation in the Δ605–612 mutant (Figure 4B), no Ser^633^ phosphorylation in the Δ626–634 mutant (Figure 4C) and no Ser^1177^ phosphorylation in the Δ14 mutant (Figure 4D). The lack of Ser^615^ phosphorylation in the Δ605–612 mutant was consistent with the hypothesis that residues 605–612 are necessary for Ser^615^ phosphorylation. It is expected that Ser^633^ and Ser^1177^ are not phosphorylated in the Δ626–634 mutant and Δ14 mutant, respectively, since these residues are no longer present. Intriguingly, the deletion of 594–604 reduced Thr^495^ phosphorylation 10-fold (Figure 4A) and increased Ser^1177^ phosphorylation 3-fold (Figure 4D) when compared with WTeNOS. Although the deletion of residues 605–612 only slightly reduced Thr^495^ phosphorylation (Figure 4A), it increased Ser^1177^ phosphorylation 2-fold (Figure 4D). Intriguingly, the extent of NO_2_^−^/NO_3_^−^ accumulation correlates proportionally with the level of Ser^1177^ phosphorylation (Figures 3A and 4D). The other two mutants, Δ626–634 and Δ14, displayed no significant differences in phosphorylation when compared with WTeNOS (Figure 4). Proper expression and equivalent loading of various eNOS proteins were confirmed by immunoblotting with an antibody against the total eNOS (Figure 4E).

### Effect of Thr^495^ phosphorylation on CaM binding

**Figure 5:**
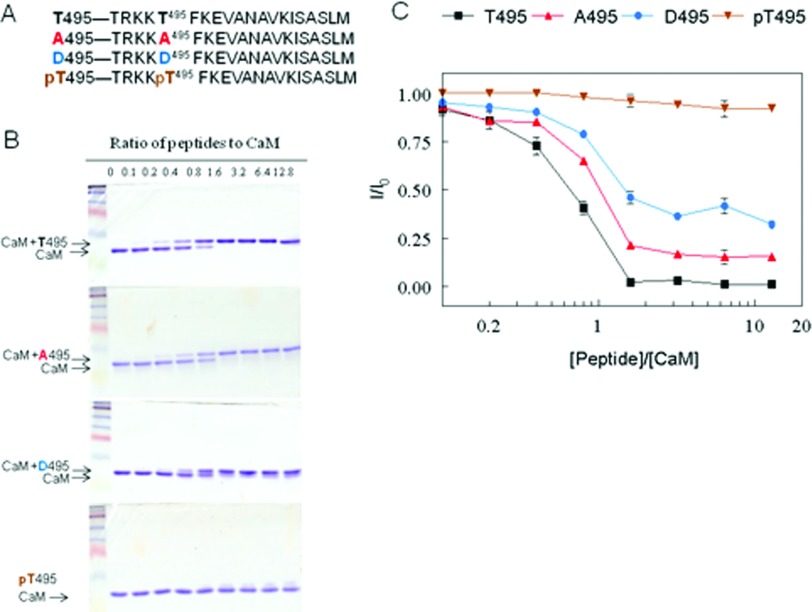
Interaction of synthetic CBD peptides with calcium/CaM (**A**) Peptide CBD sequences (residues 491–510, based on heNOS) with unmodified T495 (T495), phosphonull A substitution (A495, red colour), phosphomimetic substitution (D495, blue colour) and phosphorylated T495 (pT495, brown colour) are indicated. (**B**) The synthetic CBD variants were incubated with CaM (200 pmol) by increasing peptide:CaM molar ratios in the presence of 100 μM CaCl_2_ before electrophoresis. The samples were analysed on 18% non-denaturing gels and visualized with Coomassie Brilliant Blue R-250. Representative Coomassie Brilliant Blue-stained gels of samples containing CaM and increasing molar ratios of T495, A495, D495 and pT495 are shown. The first lane in each gel contains CaM only, i.e., CBD/CaM ratio is 0. The rest CBD/CaM ratios were 0.1:1 (2nd lane), 0.2:1 (3rd lane), 0.4:1 (4th lane), 0.8:1 (5th lane), 1.6:1 (6th lane), 3.2:1 (7th lane), 6.4:1 (8th lane) and 12.8:1 (9th lane). CBD–CaM complexes and free CaM are denoted. (**C**) The relative amount of CaM on the gel at each peptide concentration (I) was determined by densitometry and normalized to CaM in the absence of peptide (I_0_). The data are plotted as the mean I/I_0_±S.D. (*n*=4) versus peptide:CaM ratio. The graph shows CaM with increasing peptide concentration described by the following symbols: ■, T495; ▲, A495; ●, D495 and ▼, pT495.

Result from above clearly shows that the lack of phosphorylation of Thr^495^ in mutant Δ594 is followed by stimulation of Ser^1177^ phosphorylation and activation of eNOS. What needs to be explained is why a similar activation of eNOS is observed with little effect on phosphorylation of Thr^495^ in mutant Δ605. Thus, it is difficult at this stage to conclude the precise roles of Thr^495^ dephosphorylation in the activation of eNOS. Conflicting conclusions have also been drawn from earlier observations in the role of Thr^495^ phosphorylation with eNOS activation [20]. It has been reported that eNOS activity is increased by phosphorylation of Ser^1177^ despite enhanced Thr^495^ phosphorylation [43,44]. Lin et al. have shown that phosphomimetic substitution at Thr^497^ position (bovine numbering) has no effect on enzyme activity and phosphonull mutant exhibited greater enzyme activity (2–2.5-fold) than WTeNOS [45]. In contrast, Cale and Bird found that the phosphonull mutant T497A was less active than WTeNOS when stimulated by ATP and A23187 in COS-7 cell [46]. To resolve this discrepancy, we synthesized peptides representing heNOS CBD (residues 491–510) with point mutations at Thr^495^ site (Figure 5A) and determined the apparent affinity of the CBD variants to CaM. Each peptide with increasing concentrations (0–2000 pmol) was incubated with purified CaM at a fixed concentration (200 pmol) in the presence of 100 μM CaCl_2._ The mixtures were electrophoresed on non-reducing, non-denaturing gels. In this gel system, free peptides do not enter the gel because at neutral pH all peptides are positively charged, which move towards cathode. Peptide–CaM complexes migrate as higher molecular weight bands than free CaM in the presence of calcium. As shown in Figure 5(B), the unmodified peptide (Thr^495^) exhibited increases in CaM–peptide complex bands and reciprocal attenuation of free CaM bands as the peptide/CaM molar ratios increased. The phosphonull peptide (A495) and phosphomimetic peptide (D495) also showed CaM-peptide binding. The phosphorylated peptide (pThr^495^) had nearly no detectable peptide-CaM complex. The extent of CaM-peptide interaction was assessed by densitometric analysis of attenuation of free CaM bands in the presence of increasing concentrations of each peptide [47,48]. The summarized data from triplicate gels showed that the order of binding affinity for CBD variants to CaM was T495>A495>D495>pT495 (Figure 5C).

### Effects of CBD variants on eNOS activity

**Figure 6:**
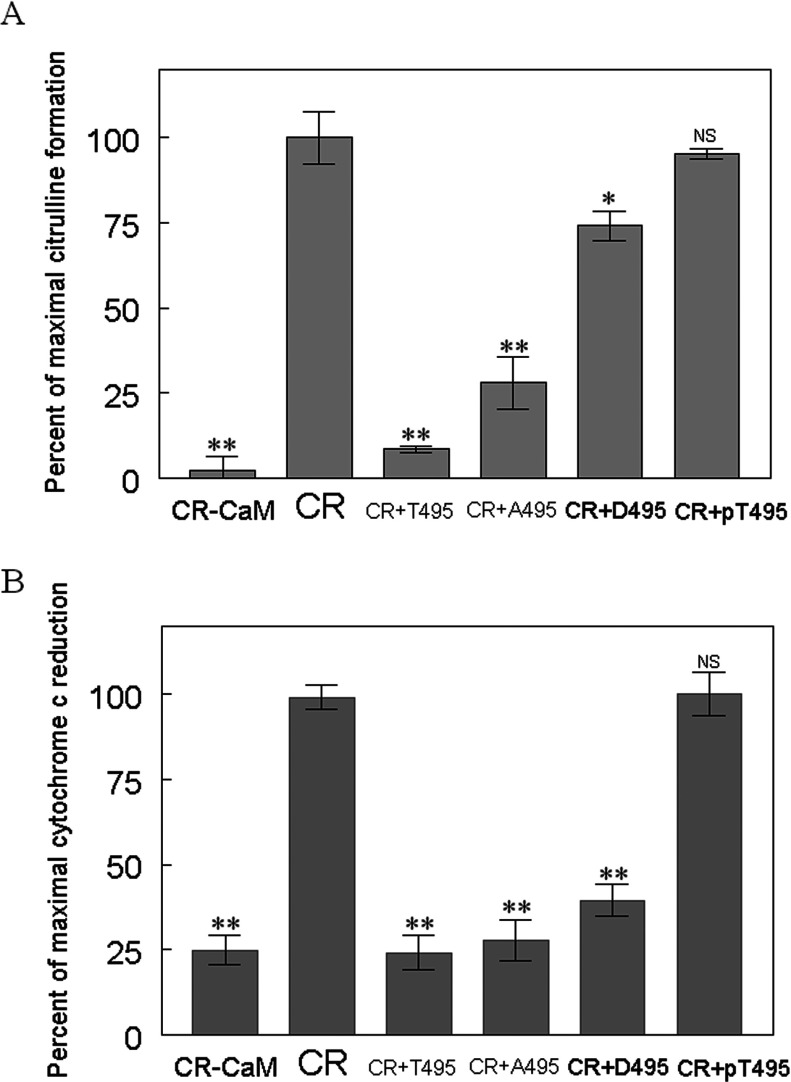
Evaluation of inhibitory potency of CBD variants on eNOS activity Modified or unmodified CBD peptides were assessed for their ability to inhibit (**A**) citrulline formation activity and (**B**) Cytochrome *c* reduction. CR denotes a complete reaction mixture; CR-CaM denotes omission of CaM from the complete reaction mixture; CR+T495 indicates the addition of 10 μM of unmodified CBD (T495) to the complete reaction mixture; CR+A495 denotes the addition of 10 μM of phosphonull CBD (A495) to the complete reaction mixture; CR+D495 indicates the addition of 10 μM of phosphomimetic CBD (D495) to the complete reaction mixture; CR+pT495 denotes the addition of 10 μM of phosphorylated CBD (pT495) to the complete reaction mixture. The complete reaction mixture for L-[^3^H]citrulline formation contains 25 mM Tris, pH 7.5, 100 mM NaCl, 0.5 μM CaM, 0.2 mM EDTA, 0.3 mM CaCl_2_, 100 μM β-NADPH, 10 μM H_4_B, 20 μM L-arginine, 1 μCi of L-[^3^H]arginine, 100 nM eNOS. The complete reaction mixture for cytochrome *c* reduction contains 25 mM Tris–HCl, pH 7.5, 100 mM NaCl, 10% glycerol, 50 μM cytochrome *c*, 0.5 μM CaM, 100 μM CaCl_2_ and 100 μM β-NADPH. The catalytic activity was normalized to give percentages relative to the reaction rate in the absence of each peptide. Each bar represents mean±S.D. of triplicate experiments. Under these conditions, the activities for eNOS bound to CaM were 12±2/min for citrulline formation, and 295±15/min for cytochrome *c* reduction. Data are presented as means±S.D.*denotes *P*<0.05, ** *P*<0.01, NS, not significant difference as compared with that in the absence of synthetic CBD peptides. Each experiment was performed in triplicate and repeated three times.

We further examined the effects of various CBD peptides on eNOS oxygenase activity by measuring the conversion of L-arginine to L-citrulline. Under the same concentration of each peptide (10 μM), the unmodified CBD (T495) inhibited L-citrulline formation by 87%. A495 and D495 inhibited citrulline formation by 70 and 20%, respectively. The phosphorylated peptides pT495 did not interfere with eNOS oxygenase activity. This inhibition was correlated with peptide-binding affinity to CaM, i.e., higher CaM-binding affinity showed more potent inhibition (Figure 6A).

To determine the various CBD peptides for their effects on the electron transfer within the eNOS reductase domain, we performed a cytochrome *c* reduction assay. An assay in the absence of CaM was carried out to serve as baseline reference. At 10 μM, unmodified CBD peptide (T495) inhibits the activity completely. Phosphonull CBD peptide (A495) and phosphomimetic CBD peptide (D495) inhibited cytochrome *c* reductase activity by 90% and 75%, respectively. In contrast, the phosphorylated CBD peptide (pT495) had virtually no inhibition on electron transfer within reductase domain. The binding affinity of various CBD peptides to CaM reflects their inhibitory potency on eNOS oxygenase activity as well as reductase activity (Figure 6B). This finding suggests that mutation of Thr^495^ to Ala/Asp cannot fully mimic the effect of phosphorylation on the structure and dynamics of eNOS.

## DISCUSSION

The eNOS function is fundamentally modulated by protein phosphorylation. Prior work has shown that the dephosphorylation and phosphorylation of Thr^495^ and Ser^1177^ are highly coordinated in response to a variety of stimuli, which has been constantly associated with enhanced NO production in the absence of extracellular calcium. We have previously found that deletions from the AIE (Δ594–604, Δ605–612 and Δ626–634) can also lead to eNOS activation at the lower level of calcium [19], implicating that the AIE might regulate Ca^2+^/CaM-dependent eNOS activation through modulating phosphorylation at Thr^495^ and Ser^1177^. Herein we established stably transfected cell lines expressing equivalent amount of WTeNOS or AIE deletion mutants in HEK-293 cells. We demonstrated that Δ594–604 and Δ605–612 constructs in non-stimulated cells displayed higher levels of NO_2_^−^/NO_3_^−^ accumulation when compared with WTeNOS and other constructs, indicating that Δ594–604 and Δ605–612 could produce NO at a resting state of calcium concentration. The amount of NO_2_^−^/NO_3_^−^ accumulation is relative to the degree of Ser^1177^ phosphorylation. More intriguingly, mutant Δ594–604 shows close correlation between Ser^1177^ phosphorylation and Thr^495^ dephosphorylation, and enhanced NO production.

Based on the rat nNOSred crystal structure [10], the AIE insert contains an α-helix (residues 840–848 equal to 610–618 heNOS), positioned between FMN-binding domain helices, and the NADPH-binding domain helix. The remaining part of AIE is not visible in the structure, reflecting the flexibility in this region. The C-terminal tail contains one highly ordered α-helical segment (CTN, residues 1397–1413 equal to 1162–1178 heNOS), which lies at a negatively charged interface between the FMN and FAD subdomains. The phosphorylatable O-γ atom of Ser^1412^ (equal to Ser^1177^ heNOS) located at the end of the CTN helix is directed towards the negatively charged residues in FMN-binding domain. Thus, phosphorylation at this residue would cause electrostatic repulsion, destabilizing the modulatory interactions between the CTN helix and AIE region, resulting in enzyme activation. The remaining part of the C-terminal tail (CTC, residues 1414–1429 equal to 1179–1194 heNOS) cannot be seen well in the structure, indicating that CTC is flexible as well [10]. In addition to its position and flexibility, the AIE insert in eNOS possesses distinctive features: it contains a canonical calcium/CaM-binding motif (residues 610–623 heNOS) and an unusual number of putative phosphorylation sites (12 serines). Phosphorylation has been reported at Ser^615^ and Ser^633^ in response to a number of stimuli, which is associated with the reduced calcium dependence for CaM binding and enzyme activation [49,50]. Given such extraordinary characteristics, it is entirely possible that the AIE insert can act as the most important crosstalk connections among various signalling pathways, including coordination of the phosphorylation state between Thr^495^ and Ser^1177^. Indeed, mutant Δ594–604 demonstrates that reduced Thr^495^ phosphorylation is associated with enhanced Ser^1177^ phosphorylation and increased NO production in the resting cell, which suggests inter-regulatory mechanisms between eNOS phosphorylation sites. Although our data demonstrate that the AIE domain is critical in regulating the phosphorylation states of eNOS, we utilized deletion mutations in our studies which can potentially generate a wide range of effects. Further studies employing point mutations are needed to validate the mechanism of this observed effect.

**Figure 7:**
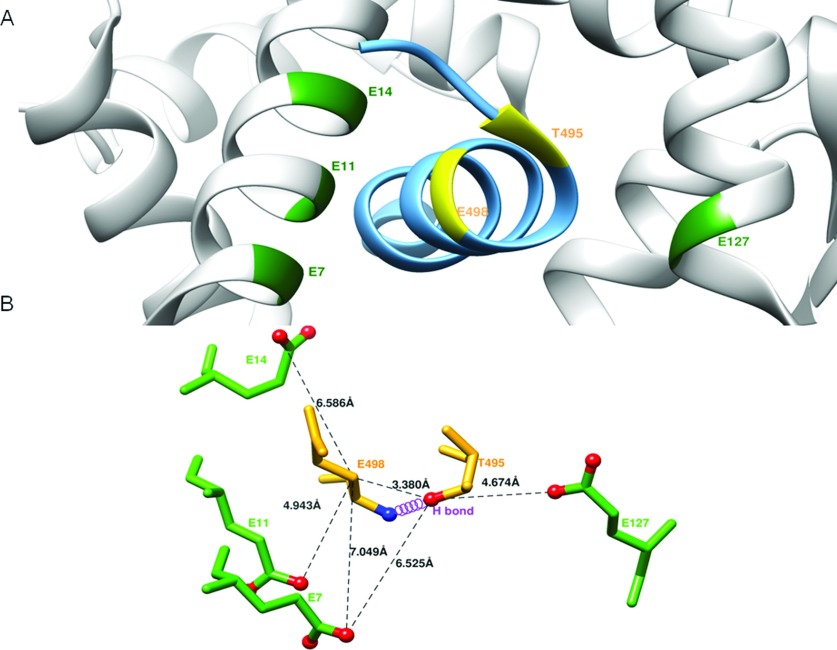
T495 phosphorylation and eNOS function (**A**) Structure of CaM and its complex with eNOS CBD (PDB accession code 1NIW). The backbone ribbons of the CBD (sky blue) and of CaM (grey) are shown. The T495 and E498 in eNOS CBD are labelled in yellow, and the neighbouring glutamate residues located on CaM in the vicinity of Thr^495^ and Glu^498^ including E7, E11, E14 and E127 are labelled in green. (**B**) Close-up view showing residues surrounding the T495 phosphorylation site. The residues as ball-and-stick representation are coloured by atom type (nitrogen, blue; oxygen, red). The hydrogen bond between Thr^495^OG1 and Glu^498^ amide N is indicated by the violet coil. The selected distances between residues are measured from the structure (1NIW) and indicated in dash lines: Thr(495)Oγ-Glu(498)Cβ, 3.38 Å; Thr(495)Oγ-Glu(7)Oε, 6.53 Å; Thr(495)Oγ-Glu(127)Oε, 4.67 Å; Glu(498)Cβ-Glu(7)Oε,7.05Å; Glu(498)Cβ-Glu(11)Oε,4.94Å; Glu(498)Cβ-Glu(14) Oε, 6.59Å.

It is generally thought that the maximal eNOS activation requires the simultaneous phosphorylation of Ser^1177^ and dephosphorylation of Thr^495^ [26]. However, this is not the case in the mutant Δ605, whose activation is observed with little effect on phosphorylation of Thr^495^. Conflicting conclusion has also been drawn from a number of studies in the role of Thr^495^ phosphorylation on eNOS function [43–46]. To this end, we used a synthesized peptide based on the heNOS CBD (residues 491–510) with point mutations at Thr^495^ site. We described the effects of Thr^495^ substitution of CBD peptides on CaM binding and eNOS activity. The result indicated that the binding affinity for CBD variants to CaM reflects their inhibitory potency on eNOS intra-domain as well as inter-domain electron transfer. The order appears to be T495>A495>D495>pT495, indicating that Thr^495^ phosphorylation reduces the CaM binding and eNOS activation. From the crystallographic structure of CaM-eNOS peptide complex [11], the side chain OG1 of T495 forms a hydrogen bond with E498 backbone N-amide in the eNOS peptide helix (Figure 7). The phosphonull mutant (T495A) without its OG1 would disrupt this hydrogen bond with the E498 backbone N, resulting in a weaker CaM binding. In the structure, T495 is surrounded by acidic residues, E7 and E127 provided by both lobes of CaM; the E498 carboxylate is surrounded by CaM E7, E11 and E14 (Figure 7) [11]. The phosphomimetic substitution or phosphorylation at T495 OG1 would not only disrupt its hydrogen bond with the E498 backbone amide, but also cause electrostatic repulsion with nearby glutamate residues of CaM. The negatively charged effect would be greater than that of phosphonull substitution. This explains why the order of CBD binding affinity to CaM as well as the inhibitory potency to eNOS activity is: unmodified CBD (T495) > phosphonull CBD (A495) > phosphoimmetic CBD (D495) > phosphorylated CBD (pT495). This finding suggests that substitutions of Thr^495^ to Ala/Asp do not reproduce full aspects of phosphorylation. Thus, the results obtained with these mimics should be interpreted with caution.

In conclusion, our data provide the first evidence that the N-terminal portion of the AIE (residues 594–604) in the FMN-binding domain could modulate the phosphorylation of the CBD and the C-terminal tail of eNOS, reflecting that the importance of the AIE in mediating CaM-dependent eNOS catalysis. Together, our results suggest that the NOS activity of the AIE deletion mutant appears to positively correlate with Ser^1177^ phosphorylation, whereas Thr^495^ phosphorylation is negatively correlated with eNOS activation under basal cellular conditions. Thus, the phosphorylation states of Ser^1177^ and Thr^495^ act in concert with AIE to control eNOS activation. Given that a variety of agonists can increase Ser^1177^ phosphorylation and decrease Thr^495^ phosphorylation and subsequent NO production, our findings that the AIE region is involved in modulating Ser^1177^ and Thr^495^ phosphorylation indicate that the AIE might participate in agonist-elicited eNOS activation through the modulation of phosphorylation. At the present time, the underlying mechanism is not clear. It is conceivable that deletion of the AIE affects the accessibility of various kinases, phosphatases, CaM or other proteins interacting with eNOS, thus affecting the phosphorylation states of Ser^1177^ and Thr^495^. This hypothesis remains to be tested.
